# Optimal Vitamin C Status is Associated with Better Physical Performance in Men and Women from the EPIC-Norfolk Cohort: A Cross-Sectional Study

**DOI:** 10.1007/s00223-026-01614-7

**Published:** 2026-10-24

**Authors:** Jamie Scott, Donnie Cameron, Max Yates, Ailsa A. Welch

**Affiliations:** 1 Norwich Medical School, University of East Anglia, Norwich, NR4 7TJ UK; 2 Department of Public Health, Norwich Medical School, University of East Anglia, Norwich, UK; 3 Department of Medical Imaging, Radboud University Medical Center, Nijmegen, The Netherlands; 4 Norwich Epidemiology Centre, Faculty of Medicine and Health Sciences, Centre for Population Health, University of East Anglia, Norwich, UK; 5 Department of Rheumatology, Norfolk and Norwich University Hospital, Norwich, UK

**Keywords:** Vitamin C, Ascorbic acid, Sarcopenia, Muscle strength, Physical performance, Ageing

## Abstract

Vitamin C may be important for maintaining skeletal muscle health and preventing sarcopenia, but most previous epidemiological studies have investigated dietary intakes rather than vitamin C status. To address this gap, this study investigated cross-sectional associations between both plasma and dietary vitamin C and muscle strength and physical performance. This study analysed data from over 5000 participants of the European Prospective Investigation into Cancer and Nutrition (EPIC)-Norfolk cohort (aged 48–92 years). Multivariable linear regression models, adjusted for lifestyle, biological, medical, and dietary covariates, were used to investigate associations between dietary (categorised as quintiles, Qs) and plasma vitamin C (low < 25μmol/L, suboptimal 25 to < 50μmol/L, optimal ≥ 50μmol/L), and handgrip strength (adjusted for height: HGS_ht_), gait speed, and 5-chair-stand time. Least square means for outcome measures were calculated from regression models. Women with optimal versus low plasma vitamin C had higher HGS_ht_ (+ 0.54 kg/m^2^, *p *= 0.009), and faster gait speed and chair-stand time (+ 0.11m/s, *p *< 0.001; − 1.03s, *p *= 0.028, respectively). Men with optimal versus low plasma vitamin C had faster gait speed and chair-stand time (+ 0.05m/s, *p *= 0.01; − 0.71s, *p *= 0.026, respectively). Compared with Q1 of dietary intake, women in Q2 and Q5 had faster gait speed (+ 0.04m/s, p < 0.01 for both); women in Q4 and Q5 had faster chair-stand times (− 0.67s, *p *= 0.002; − 0.57s, *p *= 0.013, respectively). No associations were found for dietary vitamin C in men. These findings suggest that maintaining optimal plasma vitamin C status could be important for maintaining physical function. Longitudinal and intervention studies are needed to support our findings.

**Supplementary Information:**

The online version contains supplementary material available at https://doi.org/10.1007/s00223-026-01614-7.

## Introduction

Sarcopenia is an age-related disease characterised by the progressive loss of muscle mass, muscle strength, and physical performance. It is associated with poorer quality of life, an increased risk of morbidity and mortality, and increased healthcare costs [1]. Malnutrition is a known risk factor for sarcopenia, with dietary protein the main focus of research investigating nutritional strategies for sarcopenia. However, many micronutrients also play an important role in normal muscle physiology [2]. Vitamin C (ascorbic acid), an essential nutrient in humans, is a dietary antioxidant that is important for skeletal muscle health in its role as a cofactor for several hydroxylase enzymes. These enzymes are required for the synthesis of collagen, a structural component of the layers of connective tissue surrounding muscle tissue, and carnitine, a molecule required for energy production from long-chain fatty acids [3]. One of the symptoms of vitamin C deficiency (scurvy) is muscle weakness [3].

Recent research in mice has shown that vitamin C deficiency leads to significant atrophy of skeletal muscle tissue and decreased muscle strength and physical performance, and that reversing this deficiency returns these measures to normal levels [4, 5]. In humans, observational studies have investigated associations between vitamin C and measures of muscle strength and physical performance, but most studies have focused on dietary [6–17] rather than plasma [18] vitamin C, and some include men or women only. Plasma vitamin C offers an alternative, objective measure of nutritional status that is related not only to dietary intakes, but integrates the effects of non-dietary factors, for example smoking status, or the presence of disease states or inflammation [19]. Due to the complex relationship between dietary and plasma vitamin C, it is suggested that plasma concentrations are preferable to dietary intakes as an exposure variable when investigating associations between vitamin C and health or disease outcomes [20]. However, understanding associations for dietary vitamin C is also important for the purpose of dietary guidelines and recommendations.

Previously, our research group investigated associations between plasma and dietary vitamin C and measures of fat-free mass (a surrogate marker for muscle mass) in the European Prospective Investigation of Cancer Norfolk (EPIC-Norfolk) cohort, finding positive associations between both plasma and dietary vitamin C and fat-free mass in men and women [21]. At that time, measures of muscle strength and physical performance were not collected in this cohort, but were introduced at a later stage in the study. Therefore, to address current knowledge gaps, the aim of this study is to investigate associations between both plasma and dietary vitamin C, muscle strength, and physical performance, separately in men and women from the EPIC-Norfolk cohort.

## Methods

### Study Population

Over 25,000 adults (aged 40–79 years) were recruited into the EPIC-Norfolk study from the general population of Norfolk, United Kingdom between 1993 and 1997, with participants attending an initial health check following enrolment. Subsequent follow-up occurred at four additional health checks which took place between 1998 and 2018. The current study uses data from the third health check, carried out between 2004 and 2011, which was the first health check to record handgrip strength and physical performance measures (*n* = 8623 participants, aged 48–92 years).

The EPIC-Norfolk study was granted ethical approval by the Norfolk Local Research Ethics Committee and East Norfolk and Waveney National Health Service Research Governance Committee. All participants provided written informed consent. The current (secondary data analysis) study was granted ethical approval by the University of East Anglia Faculty of Medicine and Health Sciences Research Ethics Subcommittee (ETH2324-2768).

### Blood Measurements

Plasma vitamin C and high-sensitivity C-reactive protein (hsCRP) were measured in non-fasting blood samples. For plasma vitamin C, blood samples were protected from light, and 0.5mL of plasma was stabilised in 1mL of 10% metaphosphoric acid and stored at − 70 °C. Vitamin C concentration was measured within 1 week via fluorometric assay [22]. Plasma concentrations were organised into categories for analysis. Few participants had concentrations reflective of deficiency (< 11µmol/L [23], *n* = 14), therefore, ‘deficiency’ was not included as a standalone category. Different cut-points have been used to define suboptimal concentrations, including <22.7 µmol/L, <28 µmol/L, and <50 µmol/L [24–26]. We categorised plasma concentrations as ‘low’ (< 25 µmol/L), ‘suboptimal’ (25 to <50 µmol/L), and ‘optimal’ (≥ 50 µmol/L) as a compromise between these previously-used cut-points. Serum hsCRP was assessed on an Olympus AU640 Chemistry Analyser (Olympus Diagnostics) and categorised as ‘<1mg/L’, ‘1 to 3mg/L’, and ‘>3mg/L’.

### Dietary Assessment

The EPIC-Norfolk food frequency questionnaire (FFQ) was used to assess dietary intakes of vitamin C, protein, and energy intake (EI), and nutrient intakes were calculated using FETA (the Food Frequency Questionnaire European Prospective Investigation into Cancer and Nutrition Tool for Analysis) [27]. Information on vitamin C supplement use was not available. Dietary vitamin C consumption (mg/day) was categorised into quintiles (Qs) separately for men and women for analysis. Protein intake (g/day) was calculated as a percentage of energy intake, by multiplying intake (g/day) by 4 kcal/g, dividing by EI, and multiplying by 100. Estimated energy requirement (EER) was calculated for each participant using published equations [28]. The ratio of EI to EER (EI:EER) was calculated for each participant as an estimate of dietary misreporting [29].

### Handgrip Strength and Physical Performance

Handgrip strength (HGS) was measured in each hand twice using a Smedley handheld dynamometer (Scandidact), and maximum HGS in each hand was recorded. These were summed and divided by two to calculate average HGS. When measurements were missing in one hand (*n* = 62), the maximum HGS in the opposite hand was used. HGS was adjusted for body size by dividing by height^2^ (HGS_ht_) [30]. Gait speed (m/s) was measured twice over a 4-m walking course, and the fastest gait speed was selected. Chair-stand time (s) was measured as the time taken for a participant to stand up from a chair, 5 times in a row, without using their arms for assistance. Higher values for gait speed and lower values for chair stand times reflect better physical performance.

### Other Covariates

Weight (kg) and height (m) were measured to the nearest 0.2kg and 1mm, respectively, and used to calculate body mass index (BMI; kg/m^2^). Self-reported information on ethnicity, smoking status, disease status, medication use, and physical activity was collected using a Health and Lifestyle Questionnaire. Most participants (99.7%) were of White ethnicity. Smoking status was categorised as ‘never’, ‘former’, or ‘current’. Participants self-reported whether they had ever been advised by a doctor that they had specific medical conditions (including myocardial infarction, stroke, and cancer) and any medication use (including statins, steroids, antihypertensive drugs, and medication for diabetes). Physical activity was categorised as ‘inactive’, ‘moderately inactive’, ‘moderately active’, or ‘active’, based on occupational and recreational activity levels using a validated method [31].

### Statistical Analysis

Stata statistical software version 18.5 (Stata Corp.) was used for all statistical analyses. Summary statistics for all variables are reported separately for men and women, and separately for participants with plasma and dietary vitamin C measurements. The Shapiro–Wilk test was used to assess normality of continuous variables. An independent-samples *t*-test, Mann–Whitney *U* test, or chi-squared test for proportions was used to test for differences between men and women.

Nested linear regression models were used to investigate associations between plasma or dietary vitamin C (independent variables) and HGS_ht_, gait speed, and chair-stand time (dependent variables). Plasma vitamin C was entered into regression models as a categorical variable, with ‘optimal’ chosen as the reference category, as this category had the largest number of participants. Dietary vitamin C was also treated as a categorical variable, with Q1 set as the reference category. Chair-stand time was highly skewed and therefore log-transformed for all analyses. An implausible chair-stand time of >4 min was recorded for one participant, however, all other strength and physical performance measurements for this participant were within normal ranges, so this was considered erroneous and was excluded.

In nested regression models, model 1 was unadjusted (univariate associations). Model 2 was adjusted for age (years), BMI (kg/m^2^), smoking status (never, former, current), having ever experienced cancer, stroke, or myocardial infarction, use of statins, steroids, antihypertensive medication, or medication for diabetes (all categorised yes/no), physical activity (inactive, moderately inactive, moderately active, active), and hsCRP (< 1mg/L, 1 to 3mg/L, ≥3mg/L). Models for gait speed and chair-stand time were additionally adjusted for height. Models containing dietary vitamin C were additionally adjusted for protein as a percentage of EI and dietary misreporting (EI:EER). Total EI was considered as a potential covariate in dietary vitamin C models, but was not included for three reasons: (i) due to collinearity between EI and EI:EER; (ii) as energy adjustment of dietary intakes did not improve the relationship between dietary intakes and nutritional status (plasma concentrations), and; (iii) as including either EI:EER or EI as a covariate in fully-adjusted models provided comparable results. For further explanation, and justification of the choice of covariates, please see the Supplementary Methods and Supplementary Results.

In most models, a small number of outliers were identified when reviewing linear regression diagnostic plots (*n* ≤9). Models either including or excluding these outliers were compared, and removing outliers did not impact the results, therefore, all participants were included. Least square means for outcome measures were calculated across plasma and dietary vitamin C categories from regression models. For log-transformed variables, exponentiating predicted least square means can lead to biased predictions [32], therefore, least square means of model predicted log-transformed chair-stand time were back-transformed using the following calculation:

1 $$\begin{array}{*{20}c} {E\left( {\mathrm{Y|X}} \right) = {\mathrm{e}}^{{{\mathrm{E}}({\mathrm{lnY}}|{\mathrm{X}})}} \times {\mathrm{e}}^{{{\upsigma}_{{{\mathrm{lnY}}|{\mathrm{X}}}}^{2} /2}} } \\ \end{array} $$

where E(Y|X) is the predicted chair-stand time, ${\mathrm{e}}^{{{\mathrm{E}}({\mathrm{lnY}}|{\mathrm{X}})}}$ denotes the exponentiated model-estimated log-transformed chair-stand time, and ${\mathrm{e}}^{{{\upsigma}_{{{\mathrm{lnY}}|{\mathrm{X}}}}^{2} /2}}$ is a correction factor [32], where ${\upsigma}_{{{\mathrm{lnY}}|{\mathrm{X}}}}^{2}$ reflects the variance of the errors in the model, calculated using Stata’s ‘gsem’ command [33]. All regression models were then stratified by age (< 65 and ≥65 years) to determine whether associations were present in older or younger adults. A *p* value <0.05 was considered statistically significant.

## Results

### Participant Characteristics

**Fig. 1:**
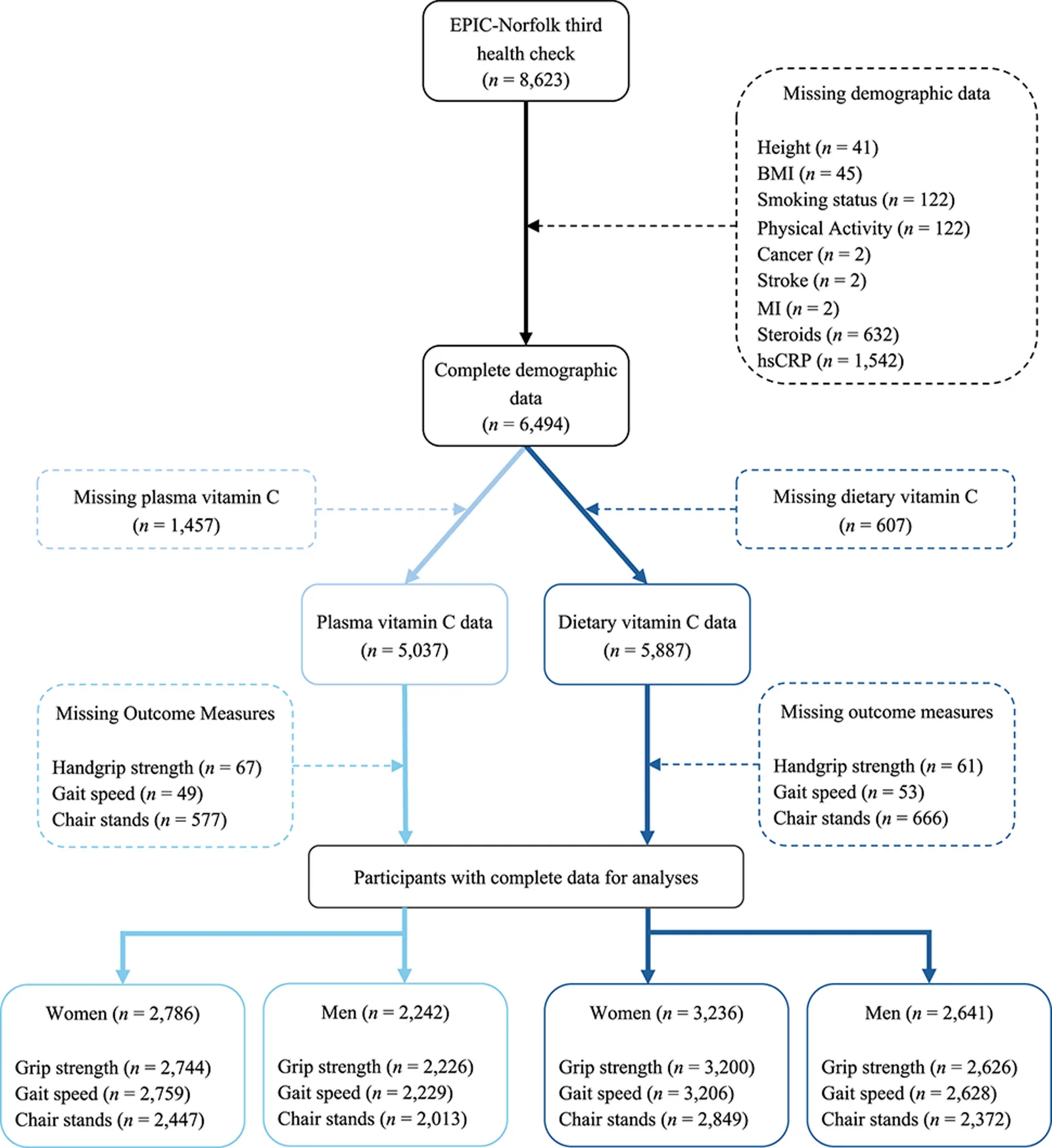
Flow chart of EPIC-Norfolk participants at the third health check with missing data, and the number of participants with complete data for analyses in the plasma vitamin C and dietary vitamin C groups. BMI: body mass index; MI: myocardial infarction; hsCRP: high sensitivity C-reactive protein

Figure 1 shows the number of participants with missing data and complete datasets for analysis. Table 1 shows participant characteristics for plasma and dietary vitamin C datasets. Men were older than women, and more likely to be physically active, whereas women were more likely to have never smoked (*p* for all <0.001). Men were more likely to have experienced stroke or myocardial infarction, whereas women were more likely to have experienced cancer (*p* for all <0.01). Medication use was higher in men (*p* for all <0.001) except for steroid use, which was similar between sexes. Women had higher plasma concentrations and dietary intakes of vitamin C (*p* for both <0.001), and men had higher HGS and HGS_ht_, and slightly faster gait speed and chair-stand times (*p* for all <0.01).

**Table 1:** Participant characteristics for the plasma vitamin C and dietary vitamin C groups, stratified by sex.^*1*^

	**Plasma vitamin C**				**Dietary vitamin C**			
	**All** (*n* = 5028)	**Women** (*n* = 2786)	**Men** (*n* = 2242)	*p *^*2*^	**All** (*n* = 5877)	**Women** (*n* = 3236)	**Men** (*n* = 2641)	*p *^*2*^
Age (years)	67.5 [62.2, 74.1]	66.6 [61.8, 73.2]	68.7 [62.9, 74.9]	<0.001*	67.8 [62.7, 74.3]	66.9 [62.2, 73.5]	69.0 [63.3, 75.1]	<0.001*
Height (m)	1.66 (0.09)	1.60 (0.06)	1.73 (0.07)	<0.001*	1.66 (0.09)	1.61 (0.06)	1.74 (0.07)	<0.001*
BMI (kg/m^2^)	26.1 [23.9, 28.8]	25.7 [23.3, 28.8]	26.5 [24.7, 28.8]	<0.001*	26.1 [23.9, 28.7]	25.7 [23.3, 28.8]	26.5 [24.6, 28.7]	<0.001*
Smoking status				<0.001*				<0.001*
Current	224 (4.5)	131 (4.7)	93 (4.2)		253 (4.3)	145 (4.5)	108 (4.1)	
Former	2338 (46.5)	1044 (37.5)	1294 (57.7)		2696 (45.9)	1171 (36.2)	1525 (57.7)	
Never	2466 (49.0)	1611 (57.8)	855 (38.1)		2928 (49.8)	1920 (59.3)	1008 (38.2)	
Physical activity				<0.001*				<0.001*
Inactive	1839 (36.6)	1016 (36.5)	823 (36.7)		2161 (36.8)	1183 (36.5)	978 (37.0)	
Moderately inactive	1452 (28.9)	908 (32.6)	544 (24.3)		1729 (29.4)	1055 (32.6)	674 (25.5)	
Moderately active	916 (18.2)	481 (17.2)	435 (19.4)		1040 (17.7)	559 (17.3)	481 (18.2)	
Active	821 (16.3)	381 (13.7)	440 (19.6)		947 (16.1)	439 (13.6)	508 (19.3)	
Cancer (yes)	530 (10.5)	325 (11.7)	205 (9.1)	0.004*	608 (10.4)	375 (11.6)	233 (8.8)	0.001*
Stroke (yes)	118 (2.4)	48 (1.7)	70 (3.1)	0.001*	123 (2.1)	50 (1.6)	73 (2.8)	0.001*
Myocardial infarction (yes)	171 (3.4)	53 (1.9)	118 (5.3)	<0.001*	209 (3.6)	67 (2.1)	142 (5.4)	<0.001*
Diabetes medication (yes)	187 (3.7)	58 (2.1)	129 (5.8)	<0.001*	221 (3.8)	78 (2.4)	143 (5.4)	<0.001*
Antihypertensive drugs (yes)	1776 (35.3)	892 (32.0)	884 (39.4)	<0.001*	2100 (35.7)	1064 (32.9)	1036 (39.2)	<0.001*
Statins (yes)	1018 (20.3)	452 (16.2)	566 (25.3)	<0.001*	1212 (20.6)	552 (17.1)	660 (25.0)	<0.001*
Steroids (yes)	208 (4.1)	106 (3.8)	102 (4.6)	0.19	241 (4.1)	128 (4.0)	113 (4.3)	0.53
hsCRP				0.15				0.06
<1 mg/L	442 (8.8)	242 (8.7)	200 (8.9)		407 (6.9)	224 (6.9)	183 (6.9)	
1–3 mg/L	3201 (63.7)	1746 (62.7)	1455 (64.9)		3880 (66.0)	2097 (64.8)	1783 (67.5)	
>3 mg/L	1385 (27.6)	798 (28.6)	587 (26.2)		1590 (27.1)	915 (28.3)	675 (25.6)	
Plasma Vitamin C (μmol/L)	62.0 [48.9, 75.8]	67.8 [54.8, 80.7]	55.8 [43.0, 68.2]	<0.001*	–	–	–	
Dietary vitamin C (mg/day)	–	–	–		129 [97, 166]	137 [104, 175]	121 [91, 155]	<0.001*
Dietary protein (g/day)	–	–	–		77.1 [64.4, 91.1]	75.5 [63.4, 89.8]	78.8 [66.1, 92.7]	<0.001*
Dietary energy (kcal/day)	–	–	–		1806 [1,491, 2178]	1704 [1425, 2048]	1929 [1603, 2.329]	<0.001*
EI:EER	–	–	–		0.82 [0.66, 1.01]	0.86 [0.70, 1.06]	0.77 [0.62, 0.95]	<0.001*
HGS (kg) ^*3*^	27.8 [22.5, 36.5]	23.3 [19.8, 26.8]	37.5 [32.0, 43.3]	<0.001*	27.5 [22.3, 36.3]	23.0 [19.5, 26.5]	37.3 [32.0, 42.8]	<0.001*
HGS_ht_ (kg/m^2^) ^*3*^	10.3 [8.6, 12.4]	9.0 [7.7, 10.3]	12.5 [10.8, 14.3]	<0.001*	10.2 [8.5, 12.3]	8.9 [7.7, 10.2]	12.4 [10.6, 14.1]	<0.001*
Gait speed (m/s) ^*4*^	1.16 [0.99, 1.31]	1.15 [0.99, 1.31]	1.17 [1.01, 1.32]	<0.001*	1.16 [1.00, 1.32]	1.15 [0.99, 1.31]	1.17 [1.02, 1.32]	<0.001*
5-Chair-stand time (s) ^*5*^	11.6 [9.5, 14.1]	11.7 [9.7, 14.3]	11.4 [9.4, 13.8]	0.002*	11.7 [9.7, 14.2]	11.8 [9.8, 14.4]	11.6 [9.5, 13.9]	0.004*

^*1*^Data presented as mean (SD), median [IQR] or n (%). * statistically significant. BMI: body mass index; EI: EER; ratio of energy intake to estimated energy expenditure; HGS: handgrip strength; HGS_ht_: height-adjusted HGS; hsCRP: high sensitivity C-reactive protein

^*2*^Differences between men and women were assessed using t-tests, Mann–Whitney U tests and Pearson chi–squared tests

^*3*^*N* = 2744 women and 2226 men in the plasma vitamin C group, and 3200 women and 2626 men in the dietary vitamin C group

^*4*^*N* = 2759 women and 2229 men in the plasma vitamin C group, and 3206 women and 2628 men in the dietary vitamin C group

^*5*^*N* = 2447 women and 2013 men in the plasma vitamin C group, and 2849 women and 2372 men in the dietary vitamin C group

### Plasma Vitamin C, Muscle Strength, and Physical Performance

**Fig. 2:**
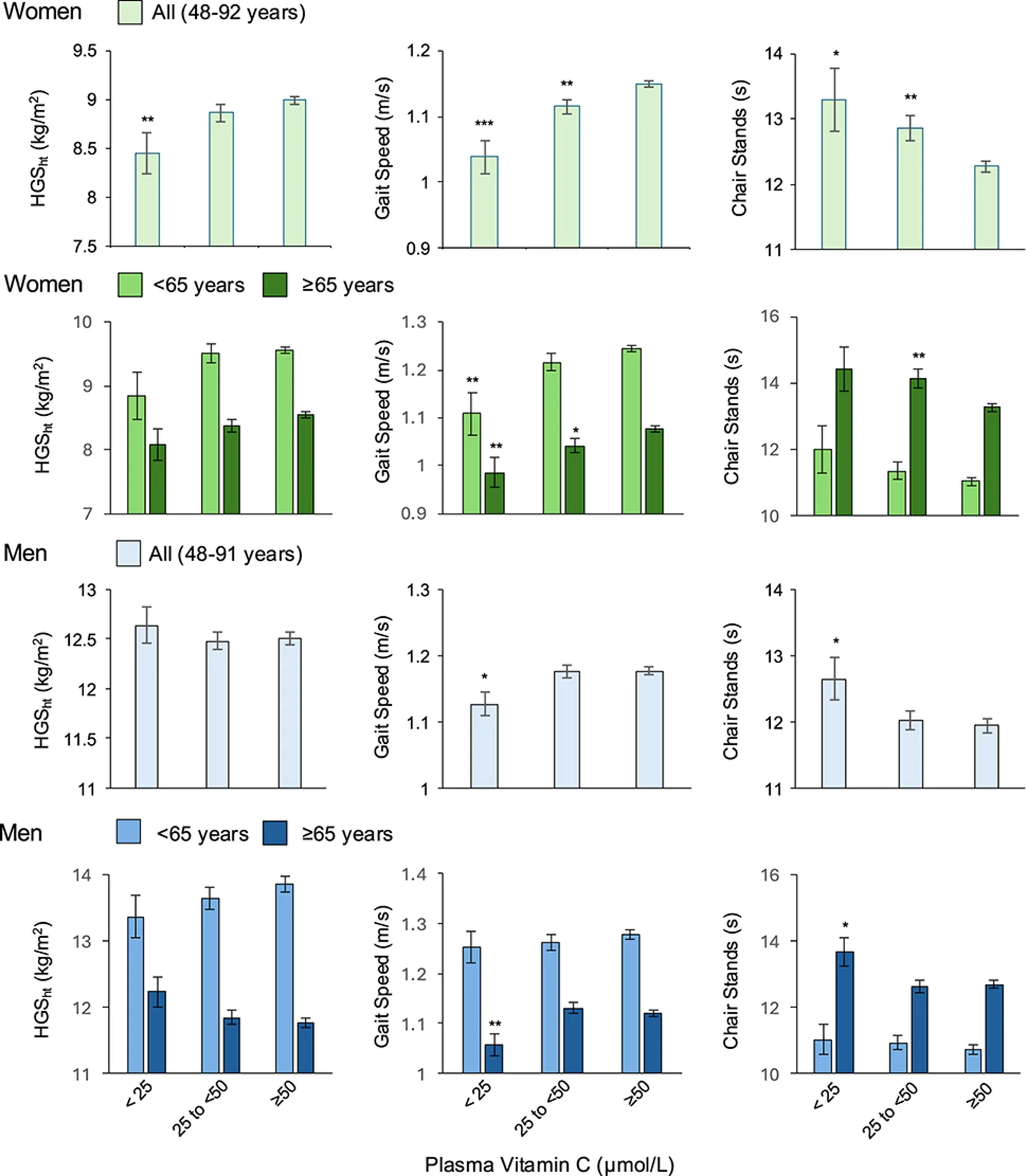
Bar graphs showing associations between plasma vitamin C categories and height-adjusted handgrip strength (HGS_ht_), gait speed and 5-chair-stand time in women (top) and men (bottom), in all participants, and in participants aged <65 and ≥65 years. Data are least square means for outcome measures (error bars represent the standard error) calculated from fully adjusted regression models, with ≥50 µmol/L plasma vitamin C set as the reference category. Models were adjusted for age, body mass index, smoking status, physical activity, cancer, stroke, myocardial infarction, use of diabetes medication, antihypertensives, statins, steroids, and high sensitivity C-reactive protein. Gait speed and chair stand models were additionally adjusted for height. For details of the number of participants included in each set of analyses, please see Supplementary Tables 1 to 3. *, **, *** significantly different from ≥50 µmol/L plasma vitamin C category. * *p*<0.05, **p<0.01, ***p<0.001

Figure 2 shows least square means for outcome measures across categories of plasma vitamin C from fully adjusted regression models, separately for men and women, and following age stratification (< 65 and ≥65 years). In all participants, after adjustment for potential confounding factors, women with optimal plasma vitamin C had higher muscle strength and better physical performance than women with low plasma vitamin C. Women with optimal plasma concentrations had 0.54kg/m^2^ higher HGS_ht_, 0.11m/s faster gait speed, and a 1.03s reduced chair-stand time (+ 6.4%, *p* = 0.009; +10.6%, *p* <0.001; − 7.7%, *p* = 0.028, respectively). When comparing women with optimal versus suboptimal plasma vitamin C, women with optimal plasma vitamin C had 0.04m/s faster gait speed and a 0.59s reduced chair-stand time (*p* = 0.004 for both). Men with optimal plasma vitamin C had a 0.05m/s faster gait speed and a 0.71s reduced chair-stand time compared to men with low plasma concentrations (+ 4.4%, *p* = 0.01; − 5.6%, *p* = 0.026, respectively). No associations were found for HGS_ht_ in men, or for gait speed or chair-stands when comparing men with optimal versus suboptimal plasma concentrations. Supplementary Table 1 shows results for unadjusted and adjusted models for all participants.

In age stratified models, the association between plasma vitamin C and HGS_ht_ in women was no longer significant, however, the difference in HGS_ht_ between women with optimal versus low plasma vitamin C was close to significance in both age groups (< 65 years, *p* = 0.053; ≥65 years, *p* = 0.058). The significant associations found between plasma vitamin C and gait speed in all women were also present in women ≥65 years (optimal versus low: +0.09m/s, +9.1%, *p* = 0.005; optimal versus suboptimal; +0.04m/s, *p* = 0.021). In women <65 years, a significant association was found when comparing women with optimal versus low status (+ 0.14m/s, +12.6%, *p* = 0.002). For chair-stand times, no association was found in women <65 years. In women ≥65 years, a significant association was found when comparing optimal with suboptimal plasma vitamin C (− 0.86s, *p* = 0.005), but no association was found when comparing optimal with low plasma concentrations. It is worth noting that there were very few women with low plasma vitamin C in both age groups (< 65 years, *n* = 26; ≥65 years, *n* = 55), which may have limited our ability to detect significant associations. In men, the significant associations found for gait speed and chair-stands persisted only in the ≥65 years age group. Older men with optimal plasma concentrations had 0.06m/s faster gait speed and a 0.99s reduced chair-stand time compared to men with low plasma concentrations (+ 5.7%, *p* = 0.007; − 7.2%, *p* = 0.02, respectively). Supplementary Tables 2 and 3 show results for unadjusted and fully adjusted age-stratified models.

### Dietary Vitamin C, Muscle Strength, and Physical Performance

**Fig. 3:**
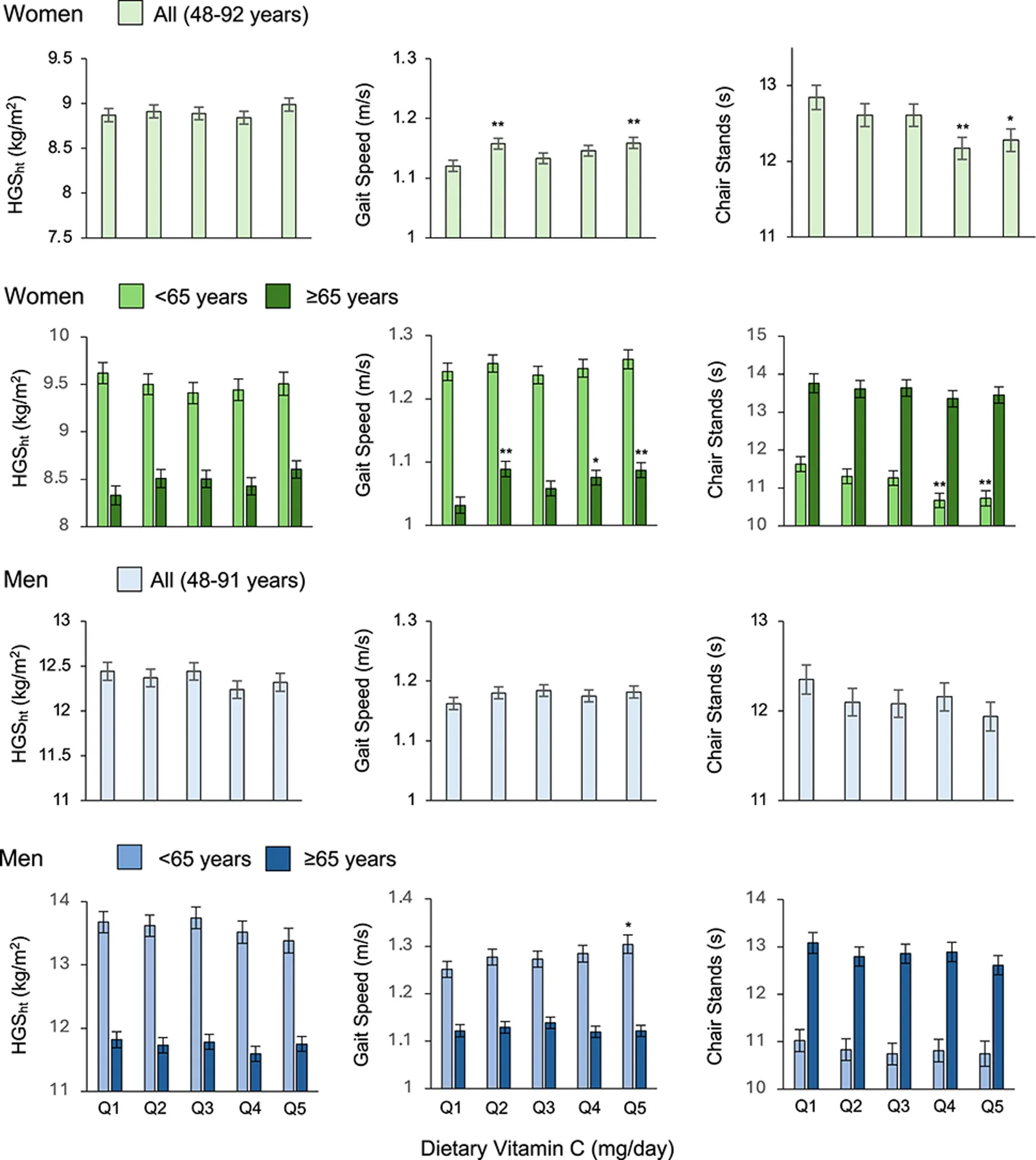
Bar graphs showing associations between quintiles of dietary vitamin C and height-adjusted handgrip strength (HGS_ht_), gait speed and 5-chair-stand time in women (top) and men (bottom), in all participants, and in participants aged <65 and ≥65 years. Data are least square means for outcome measures (error bars represent the standard error) calculated from fully adjusted regression models, with quintile 1 (Q1) of dietary vitamin C set as the reference category. Models were adjusted for age, body mass index, smoking status, physical activity, cancer, stroke, myocardial infarction, use of diabetes medication, antihypertensives, statins, steroids, high sensitivity C-reactive protein, dietary protein % of energy intake, and the ratio of energy intake to estimated energy requirements. Gait speed and chair stand models were additionally adjusted for height. For details of the number of participants included in each set of analyses, please see Supplementary Tables 4 to 6. *, ** significantly different from Q1 of dietary vitamin C. * *p*<0.05, **p<0.01

Figure 3 shows least square means for outcome measures across quintiles of dietary vitamin C from fully adjusted regression models, separately for men and women, and following age stratification (< 65 and ≥65 years). In all participants, after adjustment for potential confounding factors, women in Q2 and Q5 of dietary vitamin C intake had faster gait speed than women in Q1 (Q2: +0.04m/s, *p* = 0.004; Q5: +0.04m/s, *p* = 0.005; Q5-Q1 difference: +3.6%). Women in Q4 and Q5 had lower chair-stand times than women in Q1 of dietary intakes (Q4: − 0.67s, *p* = 0.002; Q5: − 0.57s, *p* = 0.013; Q5-Q1 difference: − 4.4%). No association was found between dietary intake of vitamin C and HGS_ht_ in women, or for HGS_ht_, gait speed, or chair-stand time in men. Full results from unadjusted and fully adjusted models are found in Supplementary Table 4.

In age-stratified models, associations between dietary vitamin C and gait speed were found in women ≥65 years (Q2: +0.06m/s, *p* = 0.001; Q4: +0.05m/s, *p* = 0.014; Q5 +0.06m/s, *p* = 0.002; Q5-Q1 difference: +5.8%), and between dietary vitamin C and chair-stand time were found in women <65 years (Q4: − 0.96s, *p* = 0.001; Q5: − 0.90s, *p* = 0.002; Q5-Q1 difference: − 7.7%). Although no associations were found between dietary vitamin C and gait speed in all men, men <65 years in Q5 versus Q1 of dietary vitamin C had 0.05m/s faster gait speed (Q5-Q1 difference: +4.0%, *p* = 0.044). Full results for unadjusted and fully-adjusted age-stratified models are found in Supplementary Table 5 and 6.

## Discussion

To our knowledge, this is the first study to investigate associations between both plasma and dietary vitamin C and muscle strength and physical performance in both men and women. We found that plasma vitamin C was associated with better muscle strength and physical performance in women, and with better physical performance in men. Dietary vitamin C was associated with better physical performance in women only, except for gait speed in younger men (< 65 years). The observed associations were generally stronger for plasma than for dietary vitamin C, and in women than in men. The inclusion of plasma vitamin C is advantageous as this offers an objective marker of vitamin C status that is not affected by different dietary assessment methods or potential dietary misreporting. Plasma vitamin C also reflects non-dietary factors that can influence nutritional status.

### Comparison With Previous Research

To our knowledge, only one previous study has investigated the association between plasma vitamin C and HGS or physical performance [18]. This cross-sectional study included older Japanese women, and found that plasma vitamin C was positively associated with HGS and standing balance, but not gait speed [18]. No studies appear to have been carried out in men or in mixed-sex cohorts.

Two prior cross-sectional studies investigated associations between dietary vitamin C and HGS in men and women together, finding no associations [6, 7]. In contrast with our findings, most prior cross-sectional studies have shown positive associations between dietary vitamin C and HGS in women [8–11]. These previous studies used different dietary assessment methods compared to the current study—one or more 24-h dietary recalls [8–10], or an FFQ plus intakes from dietary supplements [11]—which may partly explain our contrasting findings. One further cross-sectional study in women found no association between dietary vitamin C and ‘muscle quality’, measured as grip strength normalised to mean arm lean mass (grip strength/mean arm lean mass) [15]. This study also assessed dietary intakes through an FFQ, without intakes from dietary supplements [15]. In cross-sectional studies including men only, mixed findings have been reported for dietary vitamin C [8–11]. Few studies have investigated longitudinal associations between dietary vitamin C and HGS, with mixed findings. No significant associations were found in mixed sex cohorts [7, 12], or in women only [12], whereas both positive [13] and negative [12] associations have been reported in men.

Few previous studies have investigated associations between dietary vitamin C and physical performance measures. Cross-sectionally, vitamin C was positively associated with physical performance score—calculated from gait speed, chair stand and standing balance assessments—in an older Italian mixed-sex cohort [17], but no association with gait speed was found in an older Belgian mixed-sex cohort [7]. A further cross-sectional study found a positive association with chair-stands, but not gait speed, in women only [16]. Lastly, two longitudinal studies, including three mixed-sex cohorts, found no significant associations between dietary vitamin C and change in gait speed [7, 12].

Overall, evidence of an association between vitamin C and muscle strength or physical performance appears to be more consistent in women than in men. Most prior studies in this area have relied on dietary intake data to investigate these relationships, and differences in the choice of dietary assessment methods and consideration of supplement use, alongside differences in underlying cohort characteristics, may have contributed to the mixed findings. As vitamin C status is influenced by non-dietary factors, more research including plasma vitamin C is needed to help us better understand its relationship with muscle health.

### Potential Mechanisms of Vitamin C in Skeletal Muscle Health

Vitamin C is well known as a cofactor for collagen and carnitine production, both of which are important for skeletal muscle health, but it may have other mechanistic actions that are relevant to the development of sarcopenia. Mitochondria—which are found in abundance within skeletal muscle tissue—naturally produce reactive oxygen species (ROS) as part of their normal functioning, but dysfunctional mitochondria produce excess ROS that can trigger inflammation and stimulate atrophy of skeletal muscle tissue [34]. Vitamin C is an endogenous mitochondrial antioxidant that may be able to neutralise excess ROS at source, and may also regenerate vitamin E (another dietary antioxidant) within mitochondria [35]. In mice, vitamin C deficiency was shown to increase ROS levels within skeletal muscle, which returned to normal following vitamin C supplementation [4].

In vitro and ex vivo studies have shown that vitamin C may increase the activity of the PI3K/Akt/mTOR pathway—the main anabolic signalling pathway within skeletal muscle—and promote the proliferation and differentiation of skeletal muscle cells [36–39]. Studies in animals have found that vitamin C can improve muscle growth post injury [36, 37], attenuate the loss of muscle tissue during muscle atrophy [37], and influence the expression of muscle atrophy-related genes [4]. Intervention trials in humans investigating the effect of vitamin C on skeletal muscle health have generally included healthy young adults and have measured changes in outcome measures in response to exercise. In a meta-analysis of randomised clinical trials, vitamin C was found to reduce markers of inflammation and oxidative stress 1–2 h following exercise, but had no effect on muscle strength, damage or soreness [40]. A major limitation in vitamin C research in humans is the recruitment of participants who are generally healthy and likely vitamin C replete, such that further supplementation is unlikely to alter tissue concentrations, and with control groups that may already have an adequate status at baseline. These factors limit the ability to determine the effect of vitamin C in individuals with low or suboptimal vitamin C status [20].

Overall, there is evidence, albeit limited, that vitamin C plays an important role in normal muscle physiology. However, this has mostly been obtained from in vitro and animal studies, and appropriately designed studies in humans are lacking.

### Dietary Recommendations, Intakes, and Status—Are We Getting Enough to Optimise (Muscle) Health?

The UK Dietary Reference Value (DRV) for vitamin C is 40mg/day for adults, and is based on the prevention of scurvy [41]. The UK DRV is one of the lowest globally, and recommended intakes in other countries range from ~2 fold to ~5 fold higher. These increased recommendations are based not on the prevention of deficiency, but on a range of different factors; for example, maintaining an ‘adequate’ plasma concentration, or intakes that are associated with reduced risk of disease [19].

Current knowledge on the prevalence of vitamin C deficiency (plasma concentrations <11 µmol/L) is limited. A 2020 review found that most research in this area was conducted in the early 2000s or earlier, with few studies in low-middle income countries [23]. In the UK, the National Diet and Nutrition Survey (data from 2008 to 2012) found plasma vitamin C concentrations reflective of deficiency in 2.2% of adults <65 years, and 4.0% of adults ≥65 years [42]. In the cohort included in this current study, the prevalence of deficiency was very low. Nonetheless, we found positive associations between plasma vitamin C and muscle strength and physical performance.

Prior studies have shown that mean dietary intakes of vitamin C may exceed daily recommendations in individuals with deficient or suboptimal plasma concentrations [24, 25], and that around only one third of UK and 50% of US non-smoking adults can achieve an adequate plasma concentration (50 µmol/L) by meeting the country’s recommended daily intakes [43]. Current dietary recommendations, particularly in countries with the lowest recommendations, may therefore not be suitable for preventing deficiency or maintaining an optimal vitamin C status [19].

### Strengths and Limitations

To our knowledge, this study is the first to investigate associations between both plasma and dietary vitamin C and muscle strength and physical performance in a large cohort, and includes sex-stratified and age-stratified analyses. We included outcome measures that are commonly reported in the literature, allowing comparison with other studies, and adjusted our analyses for important confounding factors that are relevant to skeletal muscle health, including physical activity and dietary protein. Dietary vitamin C intakes were assessed using an FFQ, which may be less accurate than weighed food records or 7-day diet diaries [44], and we were unable to include intakes from dietary supplements. However, including plasma vitamin C allowed for associations to be explored using an objective marker of vitamin C status, although this was measured in non-fasted blood samples, which may be influenced by recent dietary intake [20]. Further, plasma vitamin C, muscle strength, and physical performance were measured concurrently. The pharmacokinetics of vitamin C are complex, and the effect of recent dietary intake on plasma concentration will depend on factors such as the dose ingested and an individual’s vitamin C status. Therefore, fasting samples are preferred when measuring vitamin C status, but are often unavailable in large cohort studies [20]. In this cohort, very few participants were vitamin C deficient, and few had low plasma concentrations; nevertheless, we observed associations between plasma vitamin C and strength and physical performance. These associations may be different—potentially stronger—in populations with higher prevalence of deficiency. Vitamin C is found in fruits and vegetables, and so higher intakes or better status may be accompanied by increased consumption of other beneficial dietary components, such as fibre and phytonutrients, and the associations found here may be partly explained by increased intakes of these dietary components. Further, circulating vitamin C may be a marker of broader healthy dietary and lifestyle patterns, which may cluster with other health behaviours, including greater physical activity. Although relevant covariates were considered in the analysis, residual cofounding and collinearity between vitamin C intake/status and other health behaviours cannot be excluded. Lastly, the cross-sectional design limits the ability to determine causal relationships, and these should be further explored in longitudinal studies.

## Conclusions

This study suggests that maintaining an optimal vitamin C status may be protective against the loss of muscle strength and physical performance during ageing, particularly in women, which may have relevance for the prevention or treatment of sarcopenia. Further observational studies are required to support our findings, particularly longitudinal studies that assess vitamin C status. Intervention studies are needed to better understand the mechanistic actions of vitamin C in skeletal muscle in humans, and to determine whether improving status is beneficial for muscle health during ageing.

## Supplementary Information

Below is the link to the electronic supplementary material.

Supplementary Material 1.

## Data Availability

Researchers wishing to access data from the EPIC-Norfolk cohort must request this directly from the EPIC-Norfolk study team. The process for requesting data from the EPIC-Norfolk study can be found on the EPIC-Norfolk website (https://www.epic-norfolk.org.uk).

## Abbreviations

EPIC-Norfolk: European Prospective Investigation into Cancer and Nutrition Norfolk cohort
BMI: Body mass index
DRV: Dietary reference value
EI: Energy intake
EER: Estimated energy requirements
FETA: Food Frequency Questionnaire European Prospective Investigation into Cancer and Nutrition Tool for Analysis
FFQ: Food frequency questionnaire
hsCRP: High-sensitivity C-reactive protein
HGS: Handgrip strength
HGS_ht_: Height-adjusted handgrip strength
Q: Quintile
ROS: Reactive oxygen species
