# Supplementary material for: Optimal Vitamin C Status is Associated with Better Physical Performance in Men and Women from the EPIC-Norfolk Cohort: A Cross-Sectional Study
Source: Calcif Tissue Int. 2026 Oct 24;117(1):168. doi: 10.1007/s00223-026-01614-7 (PMC13695595; doi:10.1007/s00223-026-01614-7)
Supplement: Supplementary file 1 [file 223_2026_1614_MOESM1_ESM.docx]

**Title:** Optimal vitamin C status is associated with better physical performance in men and women from the EPIC-Norfolk cohort: a cross-sectional study.

**Authors:** Jamie Scott, Donnie Cameron, Max Yates, Ailsa A. Welch

**Corresponding Author:** Jamie Scott, Norwich Medical School, University of East Anglia, Norwich, UK, NR4 7TJ. Email address: [J.Scott2@uea.ac.uk](mailto:J.Scott2@uea.ac.uk)

**Table of Contents**

| Supplementary Methods | Page 3 |
| --- | --- |
| Supplementary Results | Page 5 |
| Supplementary Figure 1 | Page 6 |
| Supplementary Table 1 | Page 7 |
| Supplementary Table 2 | Page 8 |
| Supplementary Table 3 | Page 9 |
| Supplementary Table 4 | Page 10 |
| Supplementary Table 5 | Page 11 |
| Supplementary Table 6 | Page 12 |
| References | Page 13 |

**Supplementary Methods**

***Adjusting for Dietary Misreporting and Energy Intake in Nutrition Epidemiology: Exploratory Analysis***

In nutrition epidemiology, two important factors should be considered when assessing associations between nutrient intakes and health outcomes: (i) dietary misreporting (the plausibility of self-reported dietary data) [1]; and (ii) adjustment for energy intake (EI) [2]. Dietary misreporting is common and can lead to inaccurate dietary intake data which may impact study results [3]. There is currently no consensus on the best method to address dietary misreporting, and several are available (Reviewed in Banna et al. [1]). This includes excluding participants with an ‘implausible’ EI, or assessing the plausibility of reported EI by comparing it to total energy expenditure (TEE) or estimated energy requirements (EER). EER reflects the daily EI predicted to maintain energy balance in healthy adults. The ratio of EI to EER (EI:EER) can be used as a covariate to statistically adjust for dietary misreporting, without the need to exclude participants based on cut-points for EI or EI:EER [1]. Therefore, in the current study, multivariable regression models for dietary vitamin C were adjusted for EI:EER.

In nutrition epidemiology, it is also generally recommended to adjust analyses for total energy intake (EI) [2]. This is important as relationships between dietary components and health or disease may be confounded by EI. Therefore, EI was considered as a potential confounding factor in regression models, but there was collinearity between EI and EI:EER (Spearman’s rho, *r_s_* = 0.87 in men and 0.91 in women), such that both variables could not be included in the same model.

Including either EI:EER or EI in fully adjusted models was explored, with comparable results found between models. For example, in women, the least square means for height-adjusted handgrip strength across quintiles of dietary vitamin C from fully-adjusted models containing EI:EER were 8.87, 8.91, 8.89, 8.84, and 8.99 kg/m^2^ respectively. For fully-adjusted models containing EI rather than EI:EER, these values were similar at 8.85, 8.91, 8.89, 8.85 and 9.01 kg/m^2^ respectively.

Adjusting for EI in the case of micronutrients has been less well explored than for macronutrients that contribute to EI. The relationship between dietary intakes of micronutrients and nutritional status (assessed using status biomarkers) may become stronger, weaker, or not substantially change following adjustment for EI. This appears to be related to how strongly intake of a given micronutrient correlates with EI, and may differ depending on the dietary assessment method and the method of energy adjustment [4,5]. Estimated EI from food frequency questionnaires (FFQs) only weakly correlates with TEE measured using doubly-labelled water [6] and heart rate monitors [7]. Jakes et al. found that body weight and physical activity were more strongly related to TEE than FFQ-estimated EI in the EPIC-Norfolk cohort, and suggested that adjustment for weight and physical activity may be more suitable than adjusting for FFQ-estimated EI [7]. However, this was questioned by Rhee et al. [4] who investigated correlations between a range of macro and micronutrient intakes (measured using an FFQ) and their respective status biomarkers following adjustment for EI, or for body weight and physical activity. They found that, following adjustment for EI, correlations were strengthened for nutrients that were more strongly correlated with EI, and advised adjusting for EI unless this is not shown to improve the relationship between intake of a nutrient and its status biomarker [4].

We therefore explored correlations, using Spearman’s rho correlation coefficients, between (i) dietary vitamin C (absolute daily intake) and energy intake, (ii) dietary vitamin C (absolute daily intake) and dietary vitamin C adjusted in three ways; adjusted for EI using the density and residual methods, and adjusted for weight and physical activity using the residual method, and; (iii) between the four dietary vitamin C variables detailed in (ii) and plasma vitamin C. To adjust for energy using the density method, dietary vitamin C intake was divided by EI. To adjust for energy using the residual method, dietary vitamin C was regressed on EI, with the model residuals reflecting energy-adjusted dietary vitamin C. To adjust for weight and physical activity, dietary vitamin C was regressed on body weight and physical activity, with the model residuals reflecting weight- and physical activity-adjusted dietary vitamin C.

Adjusting dietary intakes of vitamin C for EI, or for weight and physical activity, did not substantially alter the relationship between dietary and plasma vitamin C (please see **Supplementary Results** below). Given that these adjustments did not substantially alter the relationship between dietary intakes and plasma concentrations of vitamin C, and that regression models showed similar results whether EI or EI:EER were included as covariates, we included EI:EER, but not EI, as a confounding factor.

***Choice of Other Covariates in Statistical Models***

Multivariable regression models were adjusted for BMI and smoking status as these factors influence vitamin C status and are also associated with sarcopenia [8-11]. Models were adjusted for physical activity as this is incredibly important for skeletal muscle health [12]. We did not include race as a covariate as the vast majority (99.7%) of participants were of White ethnicity. History of disease was included as a range of disease states have been associated with sarcopenia [13-16], and may also affect vitamin C status [11]. Medication use was included as certain medications may adversely affect skeletal muscle health [17]. Models were adjusted for hsCRP as inflammation is implicated in the development of sarcopenia [18] and may also influence vitamin C status [11]. This was categorised as ‘<1mg/L’, ‘1 to 3mg/L’, and ‘>3mg/L’ based on clinical cut-points [19]. Models for gait speed and 5-chair-stand time were adjusted for height as height can influence performance on these tests [20,21]. Models for dietary vitamin C were adjusted for dietary protein (as a percentage of energy intake), as inadequate protein intake has been associated with sarcopenia [22].

**Supplementary Results**

***Correlations between Plasma and Dietary Vitamin C and Energy Intake***

Weak correlations were found between dietary vitamin C and EI in both men (*r_s_* = 0.32, *p* < 0.0001) and women (*r_s_* = 0.37, *p* <0.0001) (**Supplementary Fig. 1A**). Strong correlations were found between absolute intake of dietary vitamin C and dietary vitamin C adjusted for EI using the density method (*r_s_* = 0.75-0.77, *p* <0.0001). Very strong corelations were found between absolute intake of dietary vitamin C and dietary vitamin C adjusted for EI using the residual method (*r_s_* = 0.91-0.92, *p* <0.0001). Almost perfect correlation was found between absolute dietary vitamin C and dietary vitamin C adjusted for weight and physical activity (*r_s_*>0.99, *p* <0.0001) (**Supplementary Fig. 1A)**. Dietary intakes of vitamin C weakly corelated with plasma vitamin C in men (*r_s_* = 0.22-0.24, *p* <0.0001), and in women (*r_s_* = 0.17-0.19, *p* <0.0001), for absolute intakes, and following adjustment for EI or for weight and physical activity (**Supplementary Fig. 1B**).

**Supplementary Fig. 1** Correlations between dietary vitamin C (VC) variables, energy intake and plasma vitamin C in women and men. **A**: Correlations between absolute estimated intake of dietary vitamin C and (i) energy intake, (ii) dietary vitamin C adjusted for energy intake using the density method, (iii) dietary vitamin C adjusted for energy intake using the residual method, and (iv) dietary vitamin C adjusted for weight and physical activity (PA) using the residual method. **B**: Correlations between plasma vitamin C and (i) absolute estimated intake of dietary vitamin C, (ii) dietary vitamin C adjusted for energy intake using the density method, (iii) dietary vitamin C adjusted for energy intake using the residual method, and (iv) dietary vitamin C adjusted for weight and PA using the residual method. * p <0.0001.

**Supplementary Table 1** Cross-sectional associations between plasma vitamin C and strength and function measures.^1^

|  | **Plasma Vitamin C** | |  | **HGS_ht_ (kg/m^2^)** | |  | **Gait Speed (m/s)** | |  | **5-Chair Stand Time (s)** | |
| --- | --- | --- | --- | --- | --- | --- | --- | --- | --- | --- | --- |
|  | **(μmol/L)** | |  | **Unadjusted** *^2^* | **Adjusted** *^2,5^* |  | **Unadjusted** *^3^* | **Adjusted** *^3,5^* |  | **Unadjusted** *^4^* | **Adjusted** *^4,5^* |
|  |  |  |  |  |  |  |  |  |  |  |  |
|  | **Mean (SD)** | **Median** |  | **Mean (SE)** | **Mean (SE)** |  | **Mean (SE)** | **Mean (SE)** |  | **Mean (SE)** | **Mean (SE)** |
| **Women** |  |  |  |  |  |  |  |  |  |  |  |
| <25 μmol/L (*n* = 81) | 17.3 (5.3) | 17.3 |  | 8.19 (0.22)*** | 8.45 (0.20)** |  | 0.98 (0.03)*** | 1.04 (0.03)*** |  | 13.95 (0.54)** | 13.30 (0.48)* |
| 25 to <50 μmol/L (*n* = 439) | 40.5 (6.8) | 41.6 |  | 8.71 (0.09)** | 8.86 (0.09) |  | 1.07 (0.01)*** | 1.11 (0.01)** |  | 13.19 (0.22)*** | 12.86 (0.19)** |
| ≥50 μmol/L (*n* = 2,266) | 75.3 (17.7) | 72.2 |  | 9.03 (0.04) | 8.99 (0.04) |  | 1.16 (0.01) | 1.15 (0.005) |  | 12.19 (0.09) | 12.27 (0.08) |
| ≥50 vs <25 μmol/L difference | - | - |  | 0.84 | 0.54 |  | 0.18 | 0.11 |  | 1.76 | 1.03 |
| % difference | - | - |  | +10.3 | +6.4 |  | +18.4 | +10.6 |  | -12.6 | -7.7 |
|  |  |  |  |  |  |  |  |  |  |  |  |
| **Men** |  |  |  |  |  |  |  |  |  |  |  |
| <25 μmol/L (*n* = 159) | 17.6 (5.3) | 18.2 |  | 12.75 (0.21) | 12.63 (0.18) |  | 1.10 (0.02)*** | 1.13 (0.02)* |  | 12.63 (0.33)* | 12.64 (0.31)* |
| 25 to <50 μmol/L (*n* = 668) | 40.2 (6.7) | 40.9 |  | 12.46 (0.10) | 12.47 (0.09) |  | 1.16 (0.01)** | 1.18 (0.01) |  | 12.18 (0.16) | 12.01 (0.14) |
| ≥50 μmol/L (*n* = 1,415) | 67.6 (14.5) | 64.3 |  | 12.49 (0.07) | 12.50 (0.06) |  | 1.19 (0.01) | 1.18 (0.01) |  | 11.86 (0.10) | 11.93 (0.10) |
| ≥50 vs <25 μmol/L difference | - | - |  | 0.26 | 0.13 |  | 0.09 | 0.05 |  | 0.77 | 0.71 |
| % difference | - | - |  | -2.0 | -1.0 |  | +8.2 | +4.4 |  | -6.1 | -5.6 |

*^1^* Descriptive statistics are provided for plasma vitamin C for each plasma vitamin C category. Data for height-adjusted handgrip strength (HGS_ht_), gait speed and 5-chair stand time are presented as least square mean (standard error: SE) calculated following linear regression analysis, with ≥50 μmol/L plasma vitamin C set as the reference category. *, **, *** significant difference with ≥50μmol/L plasma vitamin C category. * *p* <0.05, ** *p* <0.01, **** p* <0.001.

*^2^* *N* = 2,744 women and 2,226 men.

*^3^* *N* = 2,759 women and 2,229 men.

*^4^ N* = 2,447 women and 2,013 men.

*^5^* Adjusted models include age, body mass index, smoking status, physical activity, history of cancer, stroke, myocardial infarction, use of diabetes medications, antihypertensives, statins, steroids and high sensitivity C-reactive protein. Gait speed and chair stand models are additionally adjusted for height.

**Supplementary Table 2** Cross-sectional associations between plasma vitamin C and strength and function measures in **women**, stratified by age (<65 years and ≥65 years).^1^

|  | **Plasma Vitamin C** | |  | **HGS_ht_ (kg/m^2^)** | |  | **Gait Speed (m/s)** | |  | **5-Chair Stand Time (s)** | |
| --- | --- | --- | --- | --- | --- | --- | --- | --- | --- | --- | --- |
|  | **(μmol/L)** | |  | **Unadjusted** *^2^* | **Adjusted** *^2,5^* |  | **Unadjusted** *^3^* | **Adjusted** *^3,5^* |  | **Unadjusted** *^4^* | **Adjusted** *^4,5^* |
|  |  |  |  |  |  |  |  |  |  |  |  |
|  | **Mean (SD)** | **Median** |  | **Mean (SE)** | **Mean (SE)** |  | **Mean (SE)** | **Mean (SE)** |  | **Mean (SE)** | **Mean (SE)** |
| **Women <65 years** |  |  |  |  |  |  |  |  |  |  |  |
| <25 μmol/L (*n* = 26) | 17.7 (5.0) | 18.0 |  | 8.65 (0.38)* | 8.84 (0.37) |  | 1.04 (0.05)*** | 1.11 (0.04)** |  | 12.59 (0.75)* | 12.01 (0.70) |
| 25 to <50 μmol/L (*n* = 160) | 40.1 (6.8) | 40.7 |  | 9.51 (0.15) | 9.53 (0.15) |  | 1.19 (0.02)** | 1.22 (0.02) |  | 11.42 (0.27) | 11.35 (0.26) |
| ≥50 μmol/L (*n* = 1,003) | 75.1 (17.4) | 72.3 |  | 9.57 (0.06) | 9.57 (0.06) |  | 1.25 (0.01) | 1.25 (0.01) |  | 11.02 (0.10) | 11.04 (0.10) |
| ≥50 vs <25 μmol/L difference | - | - |  | 0.92 | 0.73 |  | 0.21 | 0.14 |  | 1.57 | 0.97 |
| % difference | - | - |  | +10.6 | +8.3 |  | +20.2 | +12.6 |  | -12.5 | -8.1 |
|  |  |  |  |  |  |  |  |  |  |  |  |
| **Women ≥65 years** |  |  |  |  |  |  |  |  |  |  |  |
| <25 μmol/L (*n* = 55) | 17.1 (5.4) | 17.1 |  | 7.98 (0.25)* | 8.08 (0.24) |  | 0.95 (0.03)*** | 0.99 (0.03)** |  | 14.71 (0.71)* | 14.42 (0.67) |
| 25 to <50 μmol/L (*n* = 279) | 40.7 (6.8) | 41.8 |  | 8.26 (0.11)** | 8.38 (0.11) |  | 0.99 (0.02)*** | 1.04 (0.01)* |  | 14.45 (0.30)*** | 14.12 (0.28)** |
| ≥50 μmol/L (*n* = 1,263) | 75.4 (17.9) | 72.2 |  | 8.59 (0.05) | 8.55 (0.05) |  | 1.09 (0.01) | 1.08 (0.01) |  | 13.19 (0.12) | 13.26 (0.12) |
| ≥50 vs <25 μmol/L difference | - | - |  | 0.61 | 0.47 |  | 0.14 | 0.09 |  | 1.52 | 1.16 |
| % difference | - | - |  | +7.6 | +5.8 |  | +14.7 | +9.1 |  | -10.3 | -8.0 |
|  |  |  |  |  |  |  |  |  |  |  |  |

*^1^* Descriptive statistics are provided for plasma vitamin C for each plasma vitamin C category. Data for height-adjusted handgrip strength (HGS_ht_), gait speed and 5-chair stand time are presented as least square mean (standard error: SE) calculated following linear regression analysis, with ≥50 μmol/L plasma vitamin C set as the reference category. *, **, *** significant difference with ≥50μmol/L plasma vitamin C category. * *p* <0.05, ** *p* <0.01, **** p* <0.001.

*^2^* *N* = 1,176 women <65years; 1,568 women ≥65 years.

*^3^* *N* = 1,178 women <65 years; 1,581 women ≥65 years.

*^4^ N* = 1,098 women <65 years; 1,349 women ≥65 years.

*^5^* Adjusted models include age, body mass index, smoking status, physical activity, history of cancer, stroke, myocardial infarction, use of diabetes medications, antihypertensives, statins, steroids and high sensitivity C-reactive protein. Gait speed and chair stand models are additionally adjusted for height.

**Supplementary Table 3** Cross-sectional associations between plasma vitamin C and strength and function measures in **men**, stratified by age (<65 years and ≥65 years).^1^

|  | **Plasma Vitamin C** | |  | **HGS_ht_ (kg/m^2^)** | |  | **Gait Speed (m/s)** | |  | **5-Chair Stand Time (s)** | |
| --- | --- | --- | --- | --- | --- | --- | --- | --- | --- | --- | --- |
|  | **(μmol/L)** | |  | **Unadjusted** *^2^* | **Adjusted** *^2,5^* |  | **Unadjusted** *^3^* | **Adjusted** *^3,5^* |  | **Unadjusted** *^4^* | **Adjusted** *^4,5^* |
|  |  |  |  |  |  |  |  |  |  |  |  |
|  | **Mean (SD)** | **Median** |  | **Mean (SE)** | **Mean (SE)** |  | **Mean (SE)** | **Mean (SE)** |  | **Mean (SE)** | **Mean (SE)** |
| **Men <65 years** |  |  |  |  |  |  |  |  |  |  |  |
| <25 μmol/L (*n* = 58) | 17.0 (5.3) | 17.1 |  | 13.79 (0.32) | 13.36 (0.32) |  | 1.22 (0.03) | 1.25 (0.03) |  | 10.91 (0.44) | 11.02 (0.45) |
| 25 to <50 μmol/L (*n* = 228) | 40.0 (7.2) | 41.0 |  | 13.77 (0.16) | 13.64 (0.16) |  | 1.25 (0.02) | 1.26 (0.02) |  | 10.97 (0.22) | 10.92 (0.21) |
| ≥50 μmol/L (*n* = 497) | 67.0 (15.5) | 62.7 |  | 13.74 (0.11) | 13.85 (0.11) |  | 1.29 (0.01) | 1.28 (0.01) |  | 10.70 (0.14) | 10.71 (0.14) |
| ≥50 vs <25 μmol/L difference | - | - |  | 0.05 | 0.49 |  | 0.07 | 0.03 |  | 0.21 | 0.31 |
| % difference | - | - |  | -0.4 | +3.7 |  | +5.7 | +2.4 |  | -1.9 | -2.8 |
|  |  |  |  |  |  |  |  |  |  |  |  |
| **Men ≥65 years** |  |  |  |  |  |  |  |  |  |  |  |
| <25 μmol/L (*n* = 101) | 18.0 (5.4) | 18.9 |  | 12.14 (0.24) | 12.24 (0.22) |  | 1.03 (0.02)*** | 1.06 (0.02)** |  | 13.75 (0.45)** | 13.67 (0.42)* |
| 25 to <50 μmol/L (*n* = 440) | 40.3 (6.5) | 40.9 |  | 11.78 (0.12) | 11.84 (0.11) |  | 1.10 (0.01)* | 1.13 (0.01) |  | 12.90 (0.20) | 12.62 (0.19) |
| ≥50 μmol/L (*n* = 918) | 67.9 (13.9) | 65.3 |  | 11.81 (0.08) | 11.77 (0.07) |  | 1.14 (0.01) | 1.12 (0.01) |  | 12.55 (0.13) | 12.68 (0.13) |
| ≥50 vs <25 μmol/L difference | - | - |  | 0.33 | 0.47 |  | 0.11 | 0.06 |  | 1.20 | 0.99 |
| % difference | - | - |  | -2.7 | -3.8 |  | +10.7 | +5.7 |  | -8.7 | -7.2 |

*^1^* Descriptive statistics are provided for plasma vitamin C for each plasma vitamin C category. Data for height-adjusted handgrip strength (HGS_ht_), gait speed and 5-chair stand time are presented as least square mean (standard error: SE) calculated following linear regression analysis, with ≥50 μmol/L plasma vitamin C set as the reference category. *, **, *** significant difference with ≥50μmol/L plasma vitamin C category. * *p* <0.05, ** *p* <0.01, **** p* <0.001.

*^2^* *N* = 778 men <65 years; 1,448 men ≥65 years.

*^3^* *N* = 779 men <65 years; 1,450 men ≥65 years.

*^4^ N* = 753 men <65 years; 1,260 men ≥65 years.

*^5^* Adjusted models include age, body mass index, smoking status, physical activity, history of cancer, stroke, myocardial infarction, use of diabetes medications, antihypertensives, statins, steroids and high sensitivity C-reactive protein. Gait speed and chair stand models are additionally adjusted for height.

**Supplementary Table 4** Cross-sectional associations between dietary vitamin C and strength and function measures. ^1^

|  | **Vitamin C (mg/day)** | |  |  | **HGS_ht_ (kg/m^2^)** | |  | **Gait Speed (m/s)** | |  | **5-Chair Stand Time (s)** | |
| --- | --- | --- | --- | --- | --- | --- | --- | --- | --- | --- | --- | --- |
|  |  |  |  |  | **Unadjusted** *^2^* | **Adjusted** *^2,5^* |  | **Unadjusted** *^3^* | **Adjusted** *^3,5^* |  | **Unadjusted** *^4^* | **Adjusted** *^4,5^* |
|  | **Mean (SD)** | **Median** |  |  | **Mean (SE)** | **Mean (SE)** |  | **Mean (SE)** | **Mean (SE)** |  | **Mean (SE)** | **Mean (SE)** |
| **Women** |  |  |  |  |  |  |  |  |  |  |  |  |
| Q1 (*n* = 647) | 74 (17) | 77 |  |  | 8.93 (0.08) | 8.87 (0.07) |  | 1.13 (0.01) | 1.12 (0.01) |  | 12.56 (0.17) | 12.84 (0.16) |
| Q2 (*n* = 633) | 111 (8) | 111 |  |  | 8.93 (0.08) | 8.91 (0.07) |  | 1.17 (0.01)* | 1.16 (0.01)** |  | 12.47 (0.16) | 12.61 (0.15) |
| Q3 (*n* = 650) | 137 (8) | 137 |  |  | 8.88 (0.08) | 8.89 (0.07) |  | 1.13 (0.01) | 1.13 (0.01) |  | 12.61 (0.16) | 12.61 (0.15) |
| Q4 (*n* = 655) | 166 (10) | 165 |  |  | 8.84 (0.08) | 8.84 (0.07) |  | 1.14 (0.01) | 1.15 (0.01) |  | 12.27 (0.16) | 12.17 (0.15)** |
| Q5 (*n* = 651) | 239 (55) | 223 |  |  | 8.91 (0.08) | 8.99 (0.07) |  | 1.14 (0.01) | 1.16 (0.01)** |  | 12.57 (0.16) | 12.27 (0.15)* |
| Q5-Q1 difference | - | - |  |  | 0.02 | 0.12 |  | 0.01 | 0.04 |  | 0.01 | 0.57 |
| % difference | - | - |  |  | -0.2 | +1.4 |  | +0.9 | +3.6 |  | +0.1 | -4.4 |
| **Men** |  |  |  |  |  |  |  |  |  |  |  |  |
| Q1 (*n* = 525) | 64 (14) | 67 |  |  | 12.61 (0.11) | 12.44 (0.10) |  | 1.17 (0.01) | 1.16 (0.01) |  | 12.14 (0.17) | 12.35 (0.16) |
| Q2 (*n* = 524) | 96 (7) | 97 |  |  | 12.41 (0.11) | 12.37 (0.10) |  | 1.19 (0.01) | 1.18 (0.01) |  | 12.07 (0.17) | 12.10 (0.15) |
| Q3 (*n* = 532) | 120 (7) | 120 |  |  | 12.48 (0.11) | 12.44 (0.10) |  | 1.19 (0.01) | 1.18 (0.01) |  | 12.04 (0.17) | 12.08 (0.15) |
| Q4 (*n* = 529) | 147 (9) | 146 |  |  | 12.17 (0.11)** | 12.24 (0.10) |  | 1.17 (0.01) | 1.18 (0.01) |  | 12.25 (0.17) | 12.16 (0.16) |
| Q5 (*n* = 531) | 212 (62) | 195 |  |  | 12.14 (0.11)** | 12.32 (0.10) |  | 1.17 (0.01) | 1.18 (0.01) |  | 12.14 (0.17) | 11.94 (0.16) |
| Q5-Q1 difference | - | - |  |  | 0.47 | 0.12 |  | 0.0 | 0.02 |  | 0.0 | 0.41 |
| % difference | - | - |  |  | -3.7 | -1.0 |  | 0.0 | +1.7 |  | 0.0 | -3.3 |

*^1^* Descriptive statistics are provided for dietary vitamin C for each quintile of intake. Data for height-adjusted handgrip strength (HGS_ht_), gait speed and 5-chair stand time are presented as least square mean (standard error: SE) calculated following linear regression analysis, with Q1 of dietary vitamin C set as the reference category. *, ** significant difference with Q1 of dietary vitamin C intake. * *p* <0.05, ** *p* <0.01.

*^2^* *N* = 3,200 women and 2,626 men.

*^3^* *N* = 3,206 women and 2,628 men.

*^4^ N* = 2,849 women and 2,372 men.

*^5^* Adjusted models include age, body mass index, smoking status, physical activity, history of cancer, stroke, myocardial infarction, use of diabetes medications, antihypertensives, statins, steroids, high sensitivity C-reactive protein, dietary protein % of energy intake and ratio of energy intake to estimated energy requirement (EI:EER). Gait speed and chair stand models are additionally adjusted for height.

**Supplementary Table 5** Cross-sectional associations between dietary vitamin C and strength and function measures in **women**, stratified by age (<65 years and ≥65 years). ^1^

|  | **Vitamin C (mg/day)** | |  | **HGS_ht_ (kg/m^2^)** | |  | **Gait Speed (m/s)** | | | | |  | **5-Chair Stand Time (s)** | | | |  |
| --- | --- | --- | --- | --- | --- | --- | --- | --- | --- | --- | --- | --- | --- | --- | --- | --- | --- |
|  |  |  |  | **Unadjusted** *^2^* | **Adjusted** *^2,5^* |  | **Unadjusted** *^3^* | | **Adjusted** *^3,5^* | | |  | | **Unadjusted** *^4^* | | **Adjusted** *^4,5^* | |
|  | **Mean (SD)** | **Median** |  | **Mean (SE)** | **Mean (SE)** |  | **Mean (SE)** | | **Mean (SE)** | | |  | | **Mean (SE)** | | **Mean (SE)** | |
| **Women <65 years** | |  |  |  |  |  |  |  | |  |  | | | |  |  |  |
| Q1 (*n* = 293) | 74 (17) | 76 |  | 9.61 (0.11) | 9.62 (0.11) |  | 1.24 (0.01) | | 1.24 (0.01) | | |  | | 11.56 (0.20) | | 11.63 (0.20) | |
| Q2 (*n* = 279) | 111 (8) | 112 |  | 9.44 (0.11) | 9.50 (0.11) |  | 1.26 (0.01) | | 1.26 (0.01) | | |  | | 11.22 (0.20) | | 11.31 (0.19) | |
| Q3 (*n* = 263) | 136 (8) | 136 |  | 9.44 (0.11) | 9.41 (0.11) |  | 1.24 (0.01) | | 1.24 (0.01) | | |  | | 11.32 (0.20) | | 11.26 (0.19) | |
| Q4 (*n* = 260) | 165 (10) | 163 |  | 9.44 (0.12) | 9.44 (0.11) |  | 1.25 (0.01) | | 1.25 (0.01) | | |  | | 10.64 (0.20)** | | 10.67 (0.19)** | |
| Q5 (*n* = 239) | 235 (52) | 221 |  | 9.56 (0.12) | 9.51 (0.12) |  | 1.25 (0.02) | | 1.26 (0.01) | | |  | | 10.88 (0.21)* | | 10.73 (0.20)** | |
| Q5-Q1 difference | - | - |  | 0.05 | 0.11 |  | 0.01 | | 0.02 | | |  | | 0.68 | | 0.90 | |
| % difference | - | - |  | -0.5 | -1.1 |  | +0.8 | | +1.6 | | |  | | -5.9 | | -7.7 | |
| **Women ≥65 years** | |  |  |  |  |  |  |  | |  |  | | | |  |  |  |
| Q1 (*n* = 354) | 74 (17) | 78 |  | 8.38 (0.10) | 8.33 (0.10) |  | 1.04 (0.01) | | 1.03 (0.01) | | |  | | 13.48 (0.25) | | 13.76 (0.25) | |
| Q2 (*n* = 354) | 111 (8) | 111 |  | 8.53 (0.10) | 8.51 (0.10) |  | 1.09 (0.01)** | | 1.09 (0.01)** | | |  | | 13.49 (0.24) | | 13.61 (0.23) | |
| Q3 (*n* = 387) | 137 (8) | 137 |  | 8.50 (0.10) | 8.50 (0.09) |  | 1.06 (0.01) | | 1.06 (0.01) | | |  | | 13.58 (0.23) | | 13.63 (0.22) | |
| Q4 (*n* = 395) | 167 (10) | 167 |  | 8.45 (0.10) | 8.43 (0.09) |  | 1.07 (0.01)* | | 1.08 (0.01)* | | |  | | 13.51 (0.23) | | 13.35 (0.22) | |
| Q5 (*n* = 412) | 241 (57) | 224 |  | 8.53 (0.09) | 8.60 (0.09) |  | 1.08 (0.01)* | | 1.09 (0.01)** | | |  | | 13.67 (0.22) | | 13.45 (0.22) | |
| Q5-Q1 difference | - | - |  | 0.15 | 0.27 |  | 0.04 | | 0.06 | | |  | | 0.19 | | 0.31 | |
| % difference | - | - |  | +1.8 | +3.2 |  | +3.8 | | +5.8 | | |  | | +1.4 | | -2.3 | |
|  |  |  |  |  |  |  |  | |  | | |  | |  | |  | |

*^1^* Descriptive statistics are provided for dietary vitamin C for each quintile of intake. Data for height-adjusted handgrip strength (HGS_ht_), gait speed and 5-chair stand time are presented as least square mean (standard error: SE) calculated following linear regression analysis, with Q1 of dietary vitamin C set as the reference category. *, ** significant difference with Q1 of dietary vitamin C intake. * *p* <0.05, ** *p* <0.01.

*^2^* *N* = 1,325 women <65 years, 1,875 women ≥65 years.

*^3^* *N* = 1,322 women <65 years, 1,884 women ≥65 years.

*^4^ N* = 1,242 women <65 years, 1,607 women ≥65 years.

*^5^* Adjusted models include age, body mass index, smoking status, physical activity, history of cancer, stroke, myocardial infarction, use of diabetes medications, antihypertensives, statins, steroids, high sensitivity C-reactive protein, dietary protein % of energy intake and ratio of energy intake to estimated energy requirement (EI:EER). Gait speed and chair stand models are additionally adjusted for height.

**Supplementary Table 6** Cross-sectional associations between dietary vitamin C and strength and function measures in **men**, stratified by age (<65 years and ≥65 years). ^1^

|  | **Vitamin C (mg/day)** | |  | **HGS_ht_ (kg/m^2^)** | |  | **Gait Speed (m/s)** | |  | **5-Chair Stand Time (s)** | |
| --- | --- | --- | --- | --- | --- | --- | --- | --- | --- | --- | --- |
|  |  |  |  | **Unadjusted** *^2^* | **Adjusted** *^2,5^* |  | **Unadjusted** *^3^* | **Adjusted** *^3,5^* |  | **Unadjusted** *^4^* | **Adjusted** *^4,5^* |
|  | **Mean (SD)** | **Median** |  | **Mean (SE)** | **Mean (SE)** |  | **Mean (SE)** | **Mean (SE)** |  | **Mean (SE)** | **Mean (SE)** |
| **Men <65 years** | |  |  |  |  |  |  |  |  |  |  |
| Q1 (*n* = 199) | 62 (15) | 65 |  | 13.78 (0.17) | 13.68 (0.17) |  | 1.25 (0.02) | 1.25 (0.02) |  | 10.86 (0.23) | 11.02 (0.23) |
| Q2 (*n* = 186) | 96 (7) | 96 |  | 13.61 (0.17) | 13.62 (0.17) |  | 1.28 (0.02) | 1.28 (0.02) |  | 10.85 (0.24) | 10.83 (0.23) |
| Q3 (*n* = 183) | 119 (7) | 119 |  | 13.77 (0.17) | 13.74 (0.17) |  | 1.27 (0.02) | 1.27 (0.02) |  | 10.70 (0.23) | 10.74 (0.23) |
| Q4 (*n* = 174) | 146 (9) | 146 |  | 13.45 (0.18) | 13.52 (0.18) |  | 1.28 (0.02) | 1.28 (0.02) |  | 10.92 (0.25) | 10.81 (0.24) |
| Q5 (*n* = 145) | 211 (50) | 193 |  | 13.30 (0.20) | 13.38 (0.19) |  | 1.31 (0.02)* | 1.30 (0.02)* |  | 10.88 (0.27) | 10.75 (0.27) |
| Q5-Q1 difference | - | - |  | 0.48 | 0.30 |  | 0.06 | 0.05 |  | 0.02 | 0.27 |
| % difference | - | - |  | -3.5 | -2.2 |  | +4.8 | +4.0 |  | +0.2 | -2.5 |
| **Men ≥65 years** | |  |  |  |  |  |  |  |  |  |  |
| Q1 (*n* = 326) | 65 (13) | 67 |  | 11.88 (0.13) | 11.82 (0.13) |  | 1.12 (0.01) | 1.12 (0.01) |  | 12.99 (0.22) | 13.08 (0.22) |
| Q2 (*n* = 338) | 97 (7) | 97 |  | 11.74 (0.13) | 11.73 (0.12) |  | 1.14 (0.01) | 1.13 (0.01) |  | 12.79 (0.21) | 12.80 (0.20) |
| Q3 (*n* = 349) | 120 (7) | 121 |  | 11.80 (0.13) | 11.78 (0.12) |  | 1.14 (0.01) | 1.14 (0.01) |  | 12.82 (0.21) | 12.86 (0.20) |
| Q4 (*n* = 355) | 148 (10) | 146 |  | 11.55 (0.13) | 11.59 (0.12) |  | 1.12 (0.01) | 1.12 (0.01) |  | 12.98 (0.22) | 12.89 (0.21) |
| Q5 (*n* = 386) | 212 (66) | 196 |  | 11.71 (0.12) | 11.75 (0.12) |  | 1.11 (0.01) | 1.12 (0.01) |  | 12.66 (0.21) | 12.62 (0.20) |
| Q5-Q1 difference | - | - |  | 0.17 | 0.07 |  | 0.01 | 0.0 |  | 0.33 | 0.46 |
| % difference | - | - |  | -1.4 | -0.6 |  | -0.9 | 0.0 |  | -2.5 | -3.5 |

*^1^* Descriptive statistics are provided for dietary vitamin C for each quintile of intake. Data for height-adjusted handgrip strength (HGS_ht_), gait speed and 5-chair stand time are presented as least square mean (standard error: SE) calculated following linear regression analysis, with Q1 of dietary vitamin C set as the reference category. * significant difference with Q1 of dietary vitamin C intake. * *p* <0.05.

*^2^* *N* = 884 men <65 years, 1,742 men ≥65 years.

*^3^* *N* = 884 men <65 years, 1,744 men ≥65 years.

*^4^ N* = 851 men <65 years, 1,521 men ≥65 years.

*^5^* Adjusted models include age, body mass index, smoking status, physical activity, history of cancer, stroke, myocardial infarction, use of diabetes medications, antihypertensives, statins, steroids, high sensitivity C-reactive protein, dietary protein % of energy intake and ratio of energy intake to estimated energy requirement (EI:EER). Gait speed and chair stand models are additionally adjusted for height.

**References**

1. Banna, JC, McCrory, MA, Fialkowski, MK, & Boushey, C (2017) Examining Plausibility of Self-Reported Energy Intake Data: Considerations for Method Selection. Front Nutr 4:45. https://doi.org/10.3389/fnut.2017.00045

2. Willett, WC, Howe, GR, & Kushi, LH (1997) Adjustment for total energy intake in epidemiologic studies. Am J Clin Nutr 65(4 Suppl):1220S–1228S; discussion 1229S–1231S. https://doi.org/10.1093/ajcn/65.4.1220S

3. Poslusna, K, Ruprich, J, de Vries, JH, Jakubikova, M, & van't Veer, P (2009) Misreporting of energy and micronutrient intake estimated by food records and 24 hour recalls, control and adjustment methods in practice. Br J Nutr 101 Suppl 2:S73–S85. https://doi.org/10.1017/S0007114509990602

4. Rhee, JJ, Cho, E, & Willett, WC (2014) Energy adjustment of nutrient intakes is preferable to adjustment using body weight and physical activity in epidemiological analyses. Public Health Nutr 17(5):1054–1060. https://doi.org/10.1017/S1368980013001390

5. Bingham, SA, & Day, NE (1997) Using biochemical markers to assess the validity of prospective dietary assessment methods and the effect of energy adjustment. Am J Clin Nutr 65(4 Suppl):1130S–1137S. https://doi.org/10.1093/ajcn/65.4.1130S

6. Kipnis, V, Subar, AF, Midthune, D, Freedman, LS, Ballard-Barbash, R, Troiano, RP, et al. (2003) Structure of dietary measurement error: results of the OPEN biomarker study. Am J Epidemiol 158(1):14–21; discussion 22–16. https://doi.org/10.1093/aje/kwg091

7. Jakes, RW, Day, NE, Luben, R, Welch, A, Bingham, S, Mitchell, J, et al. (2004) Adjusting for energy intake--what measure to use in nutritional epidemiological studies? Int J Epidemiol 33(6):1382–1386. https://doi.org/10.1093/ije/dyh181

8. Tomlinson, DJ, Erskine, RM, Morse, CI, Winwood, K, & Onambele-Pearson, G (2016) The impact of obesity on skeletal muscle strength and structure through adolescence to old age. Biogerontology 17(3):467–483. https://doi.org/10.1007/s10522-015-9626-4

9. Hardy, R, Cooper, R, Aihie Sayer, A, Ben-Shlomo, Y, Cooper, C, Deary, IJ, et al. (2013) Body mass index, muscle strength and physical performance in older adults from eight cohort studies: the HALCyon programme. PLoS One 8(2):e56483. https://doi.org/10.1371/journal.pone.0056483

10. Degens, H, Gayan-Ramirez, G, & van Hees, HW (2015) Smoking-induced skeletal muscle dysfunction: from evidence to mechanisms. Am J Respir Crit Care Med 191(6):620–625. https://doi.org/10.1164/rccm.201410-1830PP

11. Carr, AC, & Rowe, S (2020) Factors Affecting Vitamin C Status and Prevalence of Deficiency: A Global Health Perspective. Nutrients 12(7):1963. https://doi.org/10.3390/nu12071963

12. Sanchez-Sanchez, JL, He, L, Morales, JS, de Souto Barreto, P, Jimenez-Pavon, D, Carbonell-Baeza, A, et al. (2024) Association of physical behaviours with sarcopenia in older adults: a systematic review and meta-analysis of observational studies. Lancet Healthy Longev 5(2):e108–e119. https://doi.org/10.1016/S2666-7568(23)00241-6

13. Zhang, FM, Wu, HF, Shi, HP, Yu, Z, & Zhuang, CL (2023) Sarcopenia and malignancies: epidemiology, clinical classification and implications. Ageing Res Rev 91:102057. https://doi.org/10.1016/j.arr.2023.102057

14. Su, Y, Yuki, M, & Otsuki, M (2020) Prevalence of stroke-related sarcopenia: A systematic review and meta-analysis. J Stroke Cerebrovasc Dis 29(9):105092. https://doi.org/10.1016/j.jstrokecerebrovasdis.2020.105092

15. He, N, Zhang, Y, Zhang, L, Zhang, S, & Ye, H (2021) Relationship Between Sarcopenia and Cardiovascular Diseases in the Elderly: An Overview. Front Cardiovasc Med 8:743710. https://doi.org/10.3389/fcvm.2021.743710

16. Anagnostis, P, Gkekas, NK, Achilla, C, Pananastasiou, G, Taouxidou, P, Mitsiou, M, et al. (2020) Type 2 Diabetes Mellitus is Associated with Increased Risk of Sarcopenia: A Systematic Review and Meta-analysis. Calcif Tissue Int 107(5):453–463. https://doi.org/10.1007/s00223-020-00742-y

17. Kuzuya, M (2024) Drug-related sarcopenia as a secondary sarcopenia. Geriatr Gerontol Int 24(2):195–203. https://doi.org/10.1111/ggi.14770

18. Wiedmer, P, Jung, T, Castro, JP, Pomatto, LCD, Sun, PY, Davies, KJA, et al. (2021) Sarcopenia - Molecular mechanisms and open questions. Ageing Res Rev 65:101200. https://doi.org/10.1016/j.arr.2020.101200

19. Pearson, TA, Mensah, GA, Alexander, RW, Anderson, JL, Cannon, RO, 3rd, Criqui, M, et al. (2003) Markers of inflammation and cardiovascular disease: application to clinical and public health practice: A statement for healthcare professionals from the Centers for Disease Control and Prevention and the American Heart Association. Circulation 107(3):499–511. https://doi.org/10.1161/01.cir.0000052939.59093.45

20. Dommershuijsen, LJ, Ragunathan, J, Ruiter, R, Groothof, D, Mattace-Raso, FUS, Ikram, MA, et al. (2022) Gait speed reference values in community-dwelling older adults - Cross-sectional analysis from the Rotterdam Study. Exp Gerontol 158:111646. https://doi.org/10.1016/j.exger.2021.111646

21. Zumbrunnen, V, Riegler, TF, Haile, SR, & Radtke, T (2022) The impact of seat height on 1-min sit-to-stand test performance in COPD: a randomised crossover trial. ERJ Open Res 8(3). https://doi.org/10.1183/23120541.00033-2022

22. Coelho-Junior, HJ, Calvani, R, Azzolino, D, Picca, A, Tosato, M, Landi, F, et al. (2022) Protein Intake and Sarcopenia in Older Adults: A Systematic Review and Meta-Analysis. Int J Environ Res Public Health 19(14):8718. https://doi.org/10.3390/ijerph19148718
